# Polysaccharides from *Russula*: a review on extraction, purification, and bioactivities

**DOI:** 10.3389/fnut.2024.1406817

**Published:** 2024-04-30

**Authors:** Yan Cheng, Jian Gan, Bowen Yan, Peng Wang, Hao Wu, Caoxing Huang

**Affiliations:** ^1^Co-Innovation Center of Efficient Processing and Utilization of Forest Resources, Nanjing Forestry University, Nanjing, China; ^2^State Key Laboratory of Pharmaceutical Biotechnology, Department of Sports Medicine and Adult Reconstructive Surgery, Nanjing Drum Tower Hospital, The Affiliated Hospital of Nanjing University Medical School, Nanjing, China; ^3^Department of Biomedical Engineering, School of Biomedical Engineering and Informatics, Nanjing Medical University, Nanjing, China

**Keywords:** *Russula*, polysaccharides, extraction, purification, bioactivities

## Abstract

*Russula*, a renowned edible fungus, has gained popularity as a functional food among diverse populations due to the abundant presence of amino acids, proteins, and polysaccharides. As the primary constituents of *Russula*, polysaccharides exhibit a wide range of biological properties, making them an exceptional choice for incorporation into food, medicines, and diverse biotechnological applications. This review provides a summary of the recent research on the extraction, purification, and biological applications of polysaccharides from various *Russula* spp. Currently, there are many advanced extraction technologies, such as hot water-based extraction, alkali-based extraction, ultrasonic-assisted extraction and microwave-assisted extraction. Hence, the latest progress of extraction technologies, as well as their advantages and limitations will be discusses and summarizes in this review. The separation and purification methods of polysaccharide from *Russula* were introduced, including ethanol precipitation, deproteinization and gel filtration chromatography. It also focuses on exploring the diverse bioactive capabilities of *Russula*, including anti-oxidant, anti-tumor, immunomodulatory, anti-inflammation, and anti-bacterial properties. Hence, this review aims to foster a comprehensive understanding of the polysaccharides from various *Russula* spp. and pave the way for their promising and potential future applications in the medical and functional fields.

## 1 Introduction

**Figure 1:**
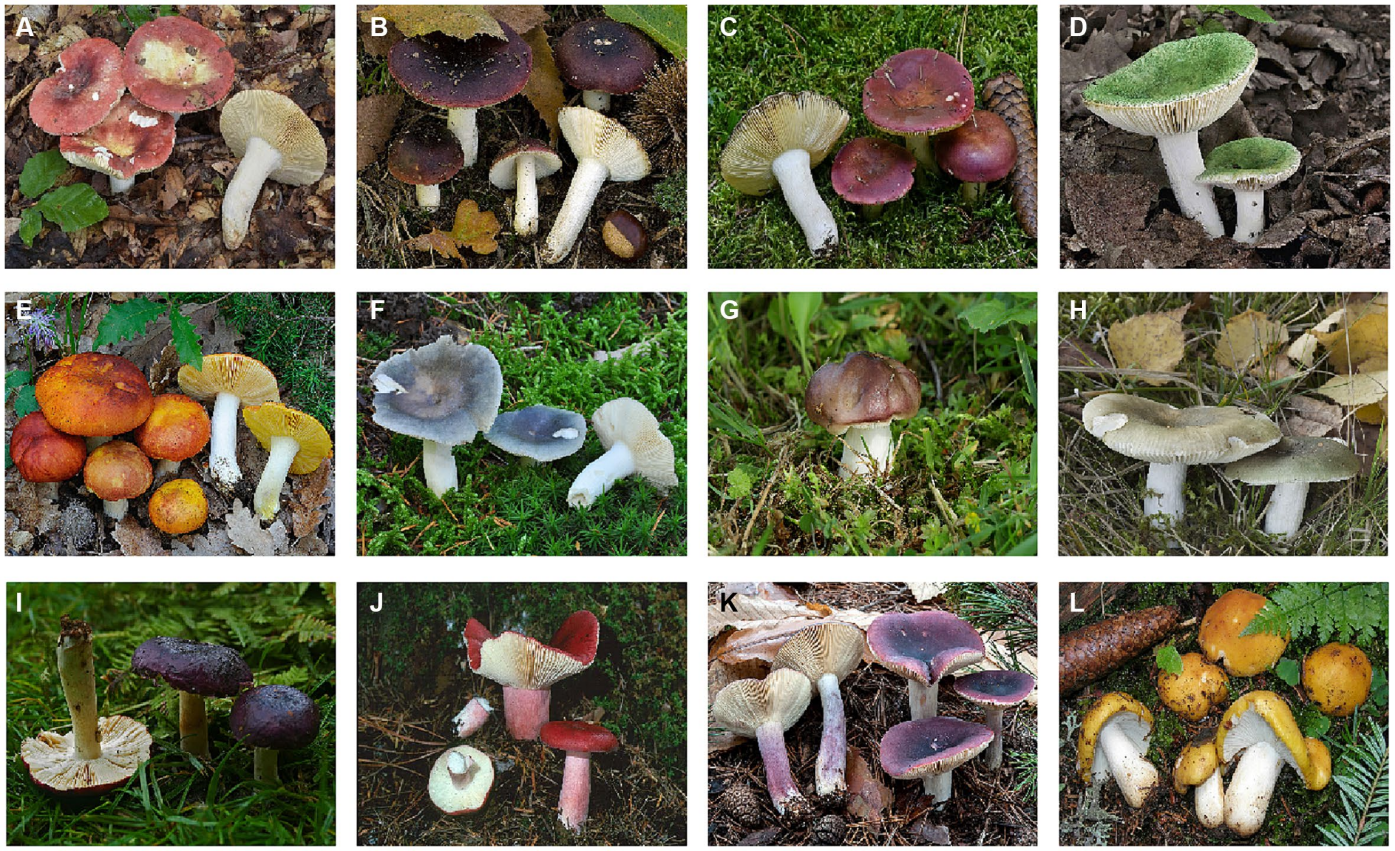
The twelve selected species of *Russula* Pers; **(A)** *Russula alutacea* Fr.; **(B)** *R. atropurpurea*; **(C)** *R. vinosa* Lindblad; **(D)** *R. virescenes* (Schaeff.) Fr.; **(E)***R. aurea* Pers.; **(F)** *R. griseocarnosa*; **(G)** *R. aeruginea* Fr.; **(H)** *R. depallens* Fr.; **(I)** *R. brunneoviolacea*; **(J)** *R. rosacea* (Pers.) Grey; **(K)** *R. sardonia* Fr.; **(L)** *R. compacta* Fros. (All edible).

*Russula* is a type of wild fungus considered to belong to a higher order, rich in phytochemicals, with a long history of use in edible and medicinal products due to its wide distribution and abundant bioactivity. Globally, the *Russula* spp. encompasses a staggering 750 varieties widely distributed in North America, Asia, Europe, and China (1). Commonly, China is home to ~100 varieties of *Russula*, mainly found in the regions of Fujian, Guangxi, Jiangxi, Yunnan, Shanxi, and others. Despite its potential applicability, large-scale artificial cultivation of *Russula* remains a challenge due to the complex task of replicating its optimal growth conditions in nature by using synthetic media as a carbon source (2, 3). In traditional Chinese medicine, *Russula* is regarded as a food supplement, highly esteemed for its ability to moisten the lungs, aid digestion, replenish blood, and exhibit anti-inflammatory and analgesic properties (4, 5). Additionally, the natural essence and inherent characteristics make *Russula* a remarkable contender for use in food- and medicine-based industries. The species of *Russula* that are traditionally used as food supplements are indicated in Figure 1.

Abundant researchers have unequivocally substantiated that the active ingredients present in *Russula*, including polysaccharides, amino acids, proteins, polyphenols, vitamins, and minerals, hold immense potential as excellent candidates for applicability in a diverse range of fields related to food, medicine, and biotechnology (6–8). Polysaccharides stand out as the primary active components in *Russula*, crucially influencing its bioactive properties. Notably, the polysaccharides in *Russula* possess a remarkable range of bioactive abilities, such as inhibiting glucosidase and amylase activities, scavenging different free radicals, and anticoagulant activities. Especially, the remarkable properties of *Russula* bestow it with the ability to inhibit the growth and proliferation of cancer cells and stimulate immunity. These underscore the significance of the potential applications of the polysaccharides from *Russula* in various fields (9, 10).

**Figure 2:**
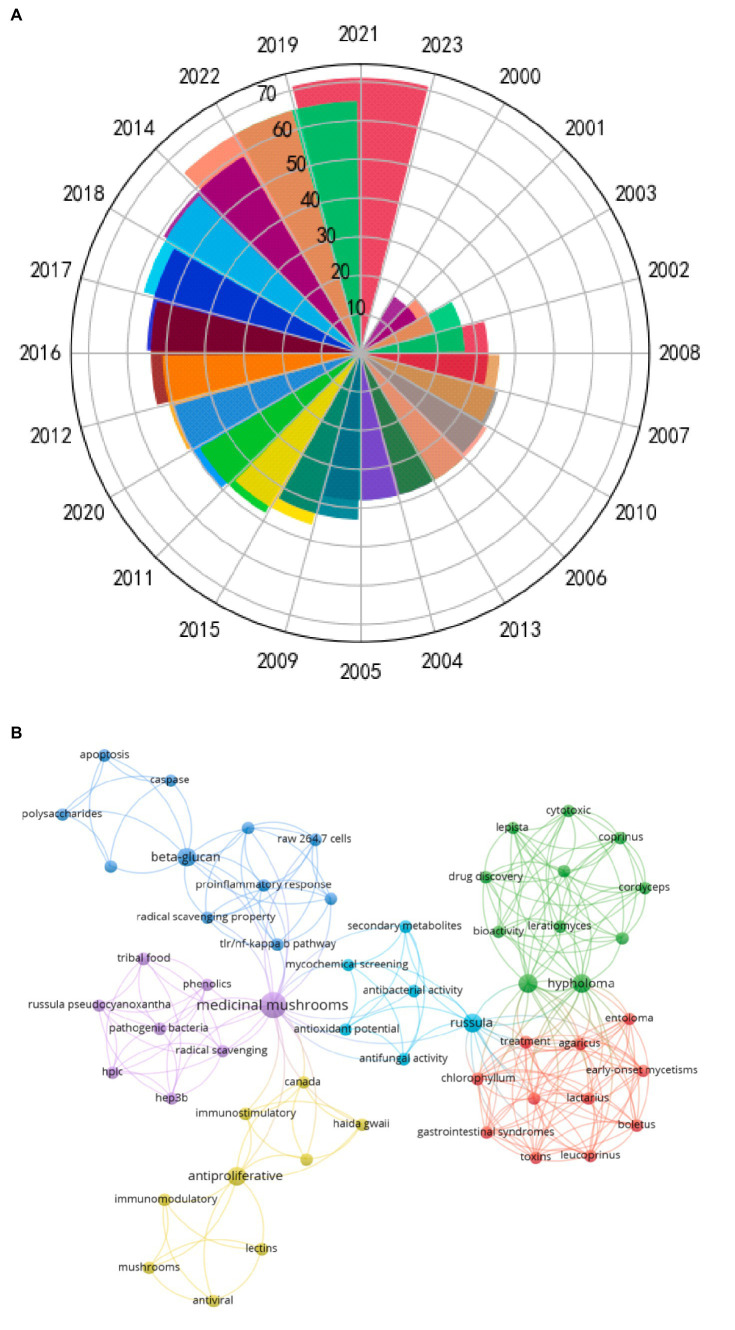
**(A)** Chronological distribution of the papers published concerning *Russula* from 2000 to 2022. **(B)** Keywords: co-occurrence mapping of *Russula* and bioactivity.

*Russula* has received extensive attention, which can be ascertained by the manifold increase in the number of papers published concerning it abound amounts of polysaccharides and bio-activities (Figure 2A). Co-Occurrence14.5 (COOC14.5) software was used to construct (document-key words) co-occurrence matrix of the full data. Through searching the Web of Science database, the search strategy was subject “*Russula*” and “biological activity,” the search time was 2000–2022, and the retrieved English journals were exported into Refworks format. Ucinet software and Netdraw software are used to generate the visual collinear network diagram of author, author and keyword. The research published over the past 20 years has indicated that *Russula* possesses good anti-oxidant (11, 12), anti-tumor (13, 14), immune-related (15, 16), anti-inflammatory (6, 17), and anti-bacterial (6, 14) activities (Figure 2B). The bioactive properties of the polysaccharides from *Russula* are intricately associated with their physicochemical and structural characteristics, such as the composition of monosaccharides, type of glycosidic bonds, and functional groups of the primary structure, much like lentinan. The presence of the β (1 → 3) glycosidic bonds in the main chain is a crucial structural determinant of their activities. For instance, the (1 → 3)-β-D-glucan present as the skeleton in polysaccharides of *Taxus* spp. exhibited potent anti-tumor activity, unlike the polysaccharides in the α-site (18). Furthermore, alterations in the spatial conformations of the polysaccharides can also exert notable effects on their functionality. Notably, H-bonds readily form between the free hydroxyl (-OH) groups on the polysaccharide chains, contributing to the dynamic nature of their functions (19). Hence, the specific structural features of the polysaccharides from *Russula* can affect its bioactivity.

The polysaccharides in *Russula* are present within the intricate matrix-associated structures of the cell walls, covalently linked with proteins and lignin molecules. Consequently, an optimal extraction process is essential for their effective isolation (10, 20). The extraction process plays a pivotal role, as the various extraction technologies can yield polysaccharides with distinct monosaccharide compositions, molecular weights (Mw), and structures, which significantly influence their biological activities, making the extraction step a critical determinant of their potential applications (20). Generally, the proposed extraction technologies include chemical-based (alkali-based) (14, 16), ultrasonic-assisted method (21–23), microwave-assisted method (24), ultra-high-pressure method (25, 26), and hot water-based extractions (10, 15, 27), which have been identified as the effective technologies to extract the polysaccharides in *Russula.* However, the proteins, lipids, and other organic molecules occurring in the cell wall and associated with the polysaccharides are co-extracted during the process (10, 20). Consequently, a purification step becomes imperative to eliminate these impurities and effectively obtain the refined polysaccharide molecules (13). In addition, this step also ensures a uniform composition and Mw of the extracted polysaccharides, which help guarantee their efficacy, potency, and safety for subsequent biomedical applications (25). Currently, most research has primarily concentrated solely on the extraction or both extraction and application of the polysaccharides from *Russula* (13). Therefore, there is a pressing need for a review encompassing the entire spectrum of the preparation processes and extraction methodologies available and exploring the potential advanced applications of these polysaccharides, thereby unlocking valuable insights into optimizing their utilities.

This review presents the latest advancements in the extraction methods and purification processes employed in preparing polysaccharides from diverse *Russula* spp. In addition, the discussions of these technologies based on their merits and limitations are included, offering valuable insights for future research. Furthermore, this review also summarizes the diverse bioactivities exhibited by the polysaccharides of *Russula*, including anti-oxidant, anti-tumor, immunomodulatory, anti-inflammation, and anti-bacterial properties. In summary, such studies would serve as a guiding resource for developing innovative technologies for extracting polysaccharides from various *Russula* spp. and facilitating their application in diverse methods of biomedical therapy.

## 2 Extraction of polysaccharides from *Russula*

Within the complex matrix of the fungal cell wall, the polysaccharides are covalently linked with other components, such as proteins and lipids, rendering them inaccessible in their natural state (25). Through extraction, these polysaccharides can be separated and isolated from the other cell wall components. Hence, extraction is a crucial step for obtaining the polysaccharides from *Russula*, enabling the analysis of their chemical composition, determination of molecular weight (Mw), and identification of functional groups (28). These characteristics significantly influence their properties and bioactivity.

**Figure 3:**
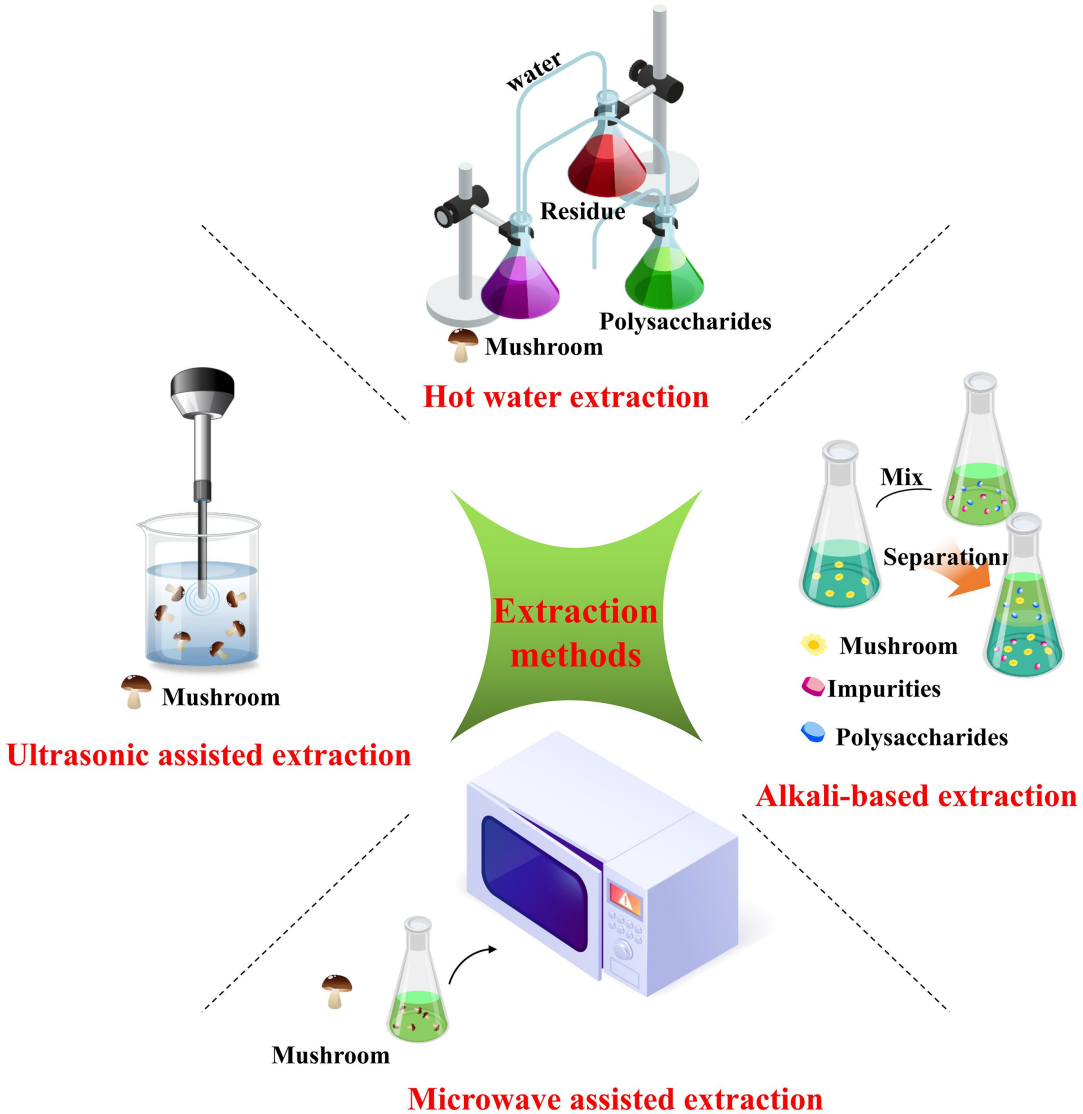
Schematic diagram of the methods used for the extraction of polysaccharides from *Russula*.

Conventional extraction methods typically rely on using hot water, acids, or alkalis under high temperature or pressure conditions (26). Specific advanced techniques for extracting polysaccharides from *Russula* have emerged as alternatives to those presently used (25). Therefore, this review summarizes the proposed technologies employed to isolate polysaccharides from *Russula*, including hot water-based, alkali-based, ultrasonic-assisted, and microwave-assisted extraction methods. These methods are presented in detail in Figure 3 for better comprehension.

### 2.1 Hot water-based extraction

Hot water-based extraction is an extensively applied technology for obtaining polysaccharides from various plants. The process involves subjecting the plant material to high temperatures for a specific duration (29). The extraction efficiency largely depends on the temperature and exposure time. The principle behind this method involves the thermal motion and high polarity of water along with the strong permeability of the cells to water causing them to expand and separate from the cell walls. As a result, water can permeate through the cell wall and into the cytoplasm, dissolving the polysaccharides and facilitating their diffusion into the extracellular solvent (30). This extraction method is recognized as a “green technology” as it does not involve using chemicals or solvents.

The extraction temperature plays a crucial role in determining the efficiency of the process. Higher temperatures can significantly improve efficiency as they facilitate the structural breakdown of the cell wall, leading to the release of polysaccharides. The yield of polysaccharides from *Russula* at various temperatures ranging from 50°C–90°C were compared, with the output reaching a peak of 4.69% at 90°C which could be attributed to their inevitable degradation at higher temperatures, resulting in a reduction of the overall yield (31). In addition, extraction time is another critical factor, with the concentration of polysaccharides obtained enhancing over time. Refined polysaccharides with yields of 1.94 and 3.58% could be extracted from *Russula* species of *R. virescens* and *R. vinosa* Lindblad at extraction times of 1–3 h, respectively (10, 27). The extraction period was observed to be directly proportional to the yield, which could be because of the improved diffusion and dissolution of polysaccharides in water. However, the extraction yield of polysaccharides from these works were still low (below 10%), which may be due to the low temperature for extraction, which should be optimized.

Moreover, the pH level also exerts a significant influence on the extraction yield of polysaccharides. Extreme pH can break the glycosidic bonds, resulting in the degradation of the polysaccharides and ultimately leading to a reduction in the yield. Therefore, maintaining an appropriate pH ensures an efficient and successful extraction. Hence, optimizing the extraction conditions is paramount, as temperature, time, solid loading, and pH can significantly influence the yield. Therefore, by carefully adjusting and fine-tuning these parameters, a higher extraction efficiency and a more desirable yield of polysaccharides from *Russula* can be achieved.

Overall, hot water-based extraction offers several significant advantages. First, its simplicity and cost-effectiveness make it an attractive option. The process does not require complex or expensive equipment and can be easily adapted for large-scale industrial production. Furthermore, hot water-based extraction is gentle, ensuring the preservation of the structural integrity and bioactivity of the polysaccharides, and does not damage these commercially valuable compounds. However, hot water-based extraction also possesses certain drawbacks. For example, a critical concern arises from its low extraction efficiency resulting in a reduced yield. Higher temperatures and extended extraction times may lead to the disruption of the structure of the polysaccharides, hindering their complete extraction. Additionally, it may not be suitable for polysaccharides with specific characteristics, such as precise solubility criteria related to pH, temperature, and ionic strength, which could affect their solubility and stability, thereby restricting the variety in their potential applications. Hence, it is needed growing-development techniques for polysaccharide production from *Russula*, which can enhance both the yield and quality of the polysaccharides for expanding their potential applications in various fields.

### 2.2 Alkali-based extraction

Chemical-assisted extraction has proven effective in enhancing the extraction efficiency of polysaccharides from various plant spp., making it a viable method for the isolation of polysaccharides from *Russula* as well (31). The alkalies used as extraction solvents, such as NaOH or KOH, has been identified with ability to effectively hydrolyze the bonds that link the polysaccharides to the proteins and lipids in the cell wall of *Russula*, thereby facilitating their separation and isolation efficiency (32). This method allows for the extraction and solubilization of polysaccharides in their free form, making them more accessible for further purification, analysis, and utilization in various applications.

Alkali-based extraction technology offers several advantages for obtaining polysaccharides from *Russula*. It can enhance the solubility of polysaccharides, leading to a higher extraction efficiency and yield. Simultaneously, the process effectively removed proteins and other impurities, producing refined polysaccharides (16, 25). Notably, the extraction efficiency for obtaining the polysaccharides from *Russula* using alkali was higher when compared to that of hot water-based extraction, enabling the elimination of low Mw compounds, including monosaccharides, disaccharides, polyphenols, and vitamins. For example, the polysaccharides from *R. senecis* were obtained using a 10% NaOH solution at 4°C for 24 h, resulting in a yield of 9.71%. The obtained polysaccharides contained various monomer sugars, such as xylose, rhamnose, mannose, and glucose, but the polysaccharide lacked a defined three-dimensional structure due to the extreme alkaline conditions (16). Compared to hot water-based extraction, alkali-based extraction of polysaccharides from *R. alatoreticula* yielded a higher proportion of β-glucans, which can further enhance its biological activity of immune response through TLR-mediated activation of the NF-κB pathway, indicating their potential immunostimulatory properties (33). In addition, it has been identified that the use of ammonium oxalate as alkali-based extraction resulted in higher polysaccharide yields and purity with lower protein contamination from *Russula*, compared with extraction processes using sodium hydroxide or sodium borohydride (34). Hence, more alkali-based extraction should be further developed aiming to improve the extraction yield of polysaccharides from *Russula.*

Based on the aforementioned discussion, it can be known that the extraction efficiency of *Russula* can be significantly improved by alkalis as solvent compared to hot water extraction. However, alkalis have the potential to partially break down the glycosidic bonds in polysaccharide molecules, resulting in hydrolysis or stripping reactions that ultimately decrease yields. Furthermore, these solvents may also lead to corrosion of extraction equipment. The extracted polysaccharide in the alkaline solution can be obtained by precipitation with ethanol. While, the remaining alkaline solution without polysaccharides is considered as wastewater, which should undergo further treatment with activated sludge or other microbial to meet the emission standards (35–37). Therefore, while alkali-based extraction may be efficient in extracting polysaccharides from *Russula*, there is a need for further exploration regarding in treating wastewater.

### 2.3 Ultrasonic-assisted extraction

Ultrasonic-assisted extraction is an innovative technology that effectively extracts bioactive compounds from plants. This method utilizes ultrasonic energy to generate acoustic cavitation and cavitation effects, leading to energy accumulation, high pressure, and temperature, which stimulate chemical reactions (38). The polysaccharides in *Russula* are primarily existed within the cell wall, but their extraction encounters hindrances such as fibers and cell mucus impeding their dissolution (21, 23). By utilizing ultrasonic waves, the extraction and separation processes are reinforced, facilitating cell wall disruption and breaking of the H-bonds, diffusion of the polysaccharides into the solvent, and increased size of the cell wall pores for the efflux of intracellular products. This breakage of the molecular cross-links enhanced the rate and efficiency of the extraction of polysaccharide (22). Consequently, ultrasonic-assisted extraction has been explored several times as a viable approach to obtain polysaccharides from *Russula* efficiently.

The application of ultrasound-based extraction has been identified as a feasible technology that could efficiently isolate polysaccharides from *Russula* (23). For instance, the conditions for the extraction of polysaccharides from *R. virescens* were further optimized, which were ultrasound at a strength of 500 W, time of 40 min, temperature of 76°C, and a water: material ratio of 31 mL/g to achieve a yield of 6.50% (39). This enhanced yield was attributed to the ultrasound-based cavitation effect, which accelerated the release of polysaccharides into the solution (39). The optimal parameters for the ultrasound-based extraction of polysaccharides from *Russula* have been investigated, which included a liquid: solid ratio of 40 mL/g, a time of 70 min, a temperature of 55°C, and ultrasound at a strength of 660 W that resulted in a remarkable yield of 6.02% which was significantly higher than that achieved through hot water-based extraction (40). Several studies have explored the optimal parameters/conditions for ultrasonic-assisted extraction of polysaccharides (23). Hence, it can be known that ultrasonic-assisted extraction can show the similar extracting performance as alkali-based extraction with better yield for polysaccharides from *Russula.*

Overall, ultrasound-based extraction technology offers numerous advantages, such as the lack of heating requirement, a shortened extraction time, and preservation of the physiological activity of the bioactive ingredients. Moreover, it overcomes limitations posed by polarity and molecular mass size, resulting in reduced time and energy consumption, and avoidance of interference from high temperatures on bioactive components. However, it is essential to exercise caution with ultrasound-based techniques, as excessive levels can disrupt the sugar chain and compromise the structural integrity of the extracted polysaccharide, which will reduce the extraction yield. Therefore, a precise and appropriate intensity of ultrasound should be applied during the extraction process (39). In summary, the cavitation and mechanical effects produced by ultrasounds enhanced the yield and bioactivity of polysaccharides from *Russula*, making it a promising processing tool for applicability in different fields.

### 2.4 Microwave-assisted extraction

In recent years, microwave-assisted extraction has gained significant traction in food science research (41). Generally, microwaves are non-ionizing radiations that induce the movement of molecules through ion migration and dipole rotation, resulting in heat generation. This process promotes cell rupture, allowing the cellular contents to spill and diffuse into the solvent. Bioactive polysaccharides can be efficiently extracted from plants using microwave-based reactors and suitable solvents. The benefits of this technique include rapid extraction, reduced solvent usage, low extraction temperature, easy separation, and high efficiency (41, 42). Consequently, microwave-assisted extraction has been explored as a viable method to obtain polysaccharides from *Russula*.

The crucial parameters to be considered for the extraction of polysaccharides from *Russula* using microwave-assisted extraction include microwave power strength, extraction time, and temperature (43, 44), which play a significant role in determining the effectiveness and efficiency of this technology. For example, the response surface method was utilized to enhance the yield of polysaccharides from *Russula* through the optimization of the different conditions set during extraction; the highest yield of 9.38% was achieved at a temperature of 48°C, pH of 5.0, microwave energy of 440 W, and time of 10 min (44). In addition, a microwave power of 170 W and an extraction time of 10 min resulted in a higher yield of 9.41% (40). A maximum yield of 14.71% was achieved under the optimized conditions of microwave energy of 515 W, pH of 3.2, extraction time of 3.1 min, and solid/liquid ratio of 1:15 g/mL (45). The results indicated that microwave-assisted extraction can also be applied to obtain the polysaccharides from *Russula*.

On comparing the yields of 1.94% (10), 3.58% (27), and 3.07% (46) obtained through hot water-based extraction, it becomes evident that microwave-assisted extraction is a more efficient technology for obtaining the polysaccharides from *Russula*. Hence, it can be known that this technology offers significant improvements in polysaccharide production compared to traditional hot water extraction. It shortens the extraction time, thus preventing the degradation of polysaccharides caused by prolonged exposure to high temperatures and avoiding the undesirable reactions like caramelization and discoloration. In addition, the polysaccharides extracted exhibited excellent quality and an intact structure. The high efficiency and reduced solvent consumption also enhance the economic benefits of using microwave-based extraction, making the industrial-scale isolation of polysaccharides from *Russula*.

In addition, enzyme- and ultra-high-pressure-assisted extractions are rapidly developing techniques that have been used to obtain polysaccharides from various edible mushrooms like *Hericium erinaceus* (47), *Ganoderma lucidum* (48), and *Lentinula edodes* (49). Enzyme-assisted extraction using pectinase and trypsin (47) effectively reduced the extraction time, minimized solvent usage, accelerated cell wall rupture, and enhanced the dissolution of polysaccharides, thereby increasing their yield from mushrooms. Ultra-high-pressure-based extraction involves subjecting a large-sized, ratio of mushroom solid to ultra-high-pressure with water, thereby allowing the dissolution of the bioactive components into the extraction solvent and maintaining a preset pressure to reach the dissolution equilibrium for the polysaccharides from different mushrooms. Currently, there is limited research on the extraction of polysaccharides from *Russula*, using these advanced methods of enzyme-assisted and ultra-high-pressure-based extraction. Hence, there is a compelling need to explore more in-depth studies and applications of these technologies in the field of the *Russula*, which can potentially lead to enhanced yields and improved quality of the obtained polysaccharides, thereby contributing to its broader utilization in various bio-field applications.

## 3 Purification of polysaccharides from *Russula*

Typically, the polysaccharides extracted from different plant spp. may be contaminated with proteins, lipids, and various other impurities, resulting in a heterogeneous distribution of sugar composition in the obtained polysaccharides (50, 51). Proteins, as the complex biomolecules, are commonly covalently or non-covalently associated with polysaccharides in *Russula*, thereby forming protein-polysaccharide complexes that might hinder their accurate characterization and potential application (52, 53). Considering the significant applications of purified polysaccharides from *Russula* in various bio-based industries, including biotechnology, nutrition, and medicine, it is the important to obtain the refined high-quality polysaccharides without impurities.

Actually, various purification techniques have been employed to obtain clarified polysaccharides from *Russula*. These methods include ethanol precipitation, deproteinization, decolorization, dialysis, and fractionation. Each of these methods plays a crucial role in eliminating impurities and enhancing the quality of the polysaccharides for diverse applications in food- and medicine-related industries (13).

### 3.1 Ethanol precipitation

**Figure 4:**
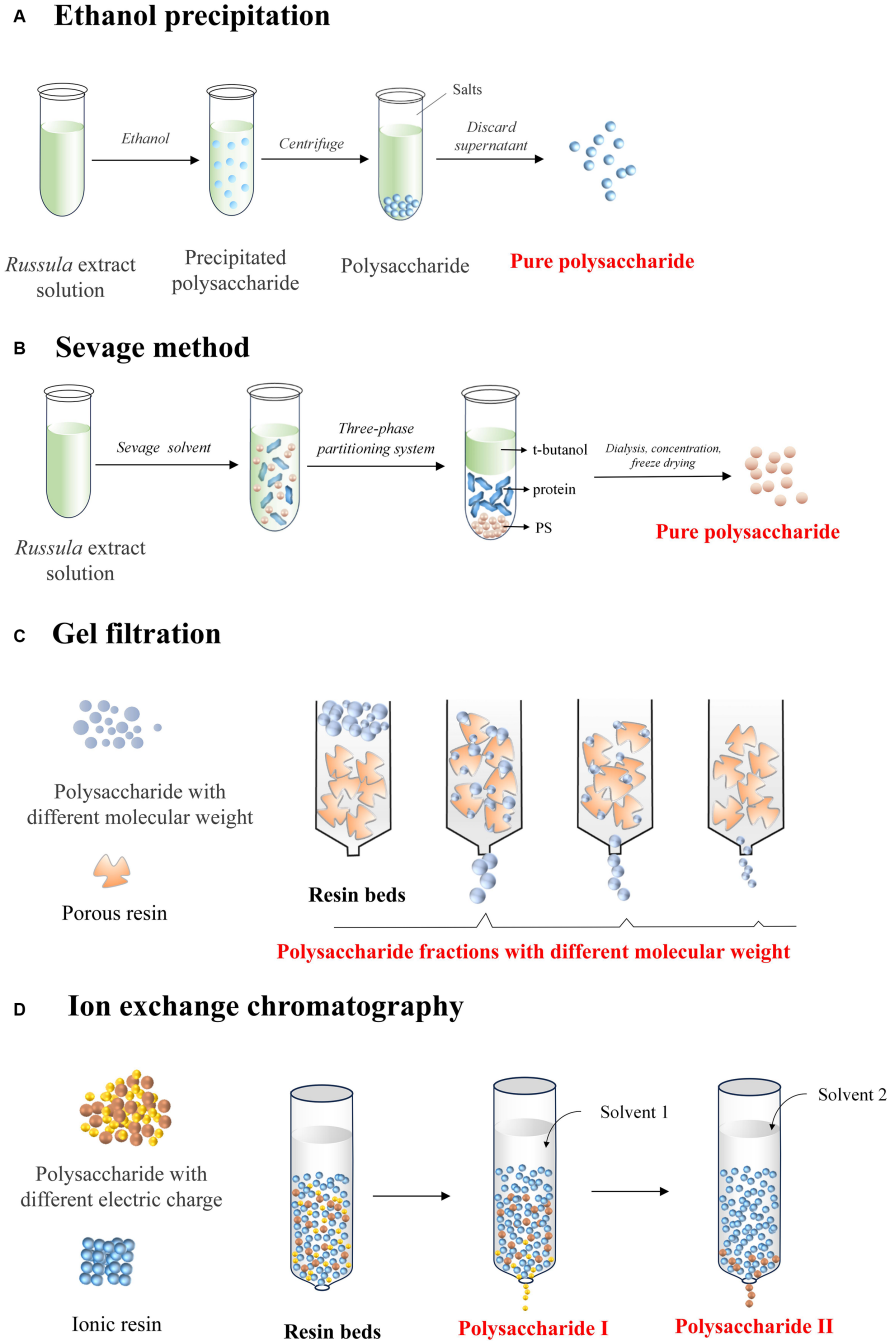
Diagram indicating the mechanisms of the various purification methods of *Russula-*derived polysaccharides using **(A)** ethanol precipitation; **(B)** Sevage method; **(C)** gel filtration; **(D)** ion exchange chromatography.

Ethanol precipitation is a widely employed technique for purifying the polysaccharides from *Russula*. It entails adding ethanol to the solution containing extracted polysaccharides and allowing it to stand at room temperature (54, 55). Subsequently, the polysaccharides can precipitate out of the solution for further easily collecting through either filtration or centrifugation, ensuring the obtaining of a refined and concentrated polysaccharide-based product (Figure 4A). This method is particularly advantageous as it eliminates small-sized impurities, such as proteins and lipids, thereby enhancing the purity of the polysaccharides extracted from *Russula*.

Ethanol precipitation has emerged as a popular one-step method for purifying the polysaccharides from various *Russula* spp. notably, *R. virescens*, *R. vinosa* Lindblad, and *R. alatoreticula*. For example, it has reported that using an ethanol precipitation gradient of 20, 40, and 65%, three fractions containing polysaccharides with different chemical structures and immunostimulatory activities were extracted from the water-extraction solution obtained from *R*. *vinosa* Lindblad (27). However, a majority of the research undertaken has primarily focused on applying the one-step precipitation method using ethanol in concentrations ranging from 75–90% to obtain relatively unadulterated polysaccharides from *Russula* spp. of *R. vinosa* (56), *R. senecis* (16), *R. virescens* (10), and *R. alutacea* (17). In gradient ethanol-based precipitation, a sequence of progressively increasing concentrations of ethanol is added to the extraction solution, allowing the polysaccharides to precipitate until the desired level of separation is gradually achieved. A single ethanol concentration is added to the solution in one-step ethanol-based precipitation, and the precipitated polysaccharides are collected. Although the latter offers greater efficiency in obtaining higher-quality polysaccharides compared to the former, it may not provide the same level of control over the yield. In summary, both methods are viable for obtaining unmixed polysaccharides from *Russula* after extraction.

Overall, ethanol precipitation offers several advantages for use with *Russula*, including simplicity, high yield, and effective removal of impurities. However, the main drawback lies in the lack of precision, as the yield can vary depending on the concentration of ethanol used, leading to the extraction of polysaccharides with varying types of bioactivities. Thus, optimization is necessary to achieve the desired polysaccharides from *Russula*. Furthermore, low-Mw polysaccharides might not readily precipitate with ethanol leading to inefficient isolation. Hence, scaling up the process can be challenging due to the increased ethanol consumption when working with large quantities.

### 3.2 Deproteinization

Deproteinization is a principal technique to separate proteins bonded with the various polysaccharides obtained from different plant spp. Proteins are often isolated as impurities during polysaccharide extraction due to their abundance in the cells of organisms (Figure 4B) (57, 58). Failure to remove these proteins can disrupt the subsequent analysis and reduce applicability in different bio-fields. The deproteinization process relies on leveraging the unique chemical properties of proteins, such as charge and hydrophilicity, to isolate them from the other components through the use of distinct separation methods (59, 60).

Several studies have explored the purification of polysaccharides from *Russula* by applying deproteinization technology. For example, the precipitate obtained from the polysaccharide-containing solution from *R. virescens* was dissolved in 300 mL of water, subjected to 20 rounds of deproteinization using the Sevage method, and then extensively dialyzed in double-distilled water for three days, resulting in the isolation of two different water-soluble, purified polysaccharides with Mw of 3.1 × 10^5^ and 4.2 × 10^5^ Da, respectively (10). Similarly, a purified polysaccharide with an average Mw of 1029.7 kDa and remarkable anti-inflammatory properties was successfully obtained from the water extraction solution of *R. alutacea* using 10 repetitions of the Sevage method (17). Likewise, a purified polysaccharide with an Mw of 3,900 kDa was obtained from the water extraction solution of *R. virescens* by combining the Sevage method with exhaustive dialysis using water for 48 h (61). Overall, these studies suggest that deproteinization is an efficient method for obtaining unadulterated polysaccharides from water-soluble extracts of *Russula*, although this technique has not been widely explored with other methods employed for the extraction of polysaccharides of *Russula.*

Generally, deproteinization offers the advantage of removing proteins from the solution, thereby enhancing the precision of the subsequent analysis. Nevertheless, this technique may lead to affect the structure and yield of polysaccharides from *Russula*. As a result, the bioactivities and functionality of *Russula*-derived polysaccharides may also be impacted. Hence, a careful consideration of the trade-offs is necessary when employing deproteinization. Hence, investigating the impact of deproteinization on the structural and functional properties of *Russula*-derived polysaccharides would be valuable in understanding its potential applications in various industries. Furthermore, research on developing novel deproteinization techniques that are more selective and less damaging to the structure of the polysaccharide can be pursued to enhance the overall efficiency and effectiveness of the process. Moreover, studying the effects of deproteinization on the bioactivities of *Russula*-derived polysaccharides in various biological systems would provide insights into its potential therapeutic uses.

### 3.3 Gel filtration chromatography

Gel filtration chromatography (GFC), also known as size exclusion chromatography, is commonly applied for the purification and separation of polysaccharides from various plants based on their Mw. Since polysaccharides can vary significantly in their Mw, GFC possess the ability to isolate polysaccharides of a specific Mw and eliminate others (62, 63). This technique is handy for purifying the polysaccharide fractions from *Russula* with known Mw, ensuring their accurate and reliable characterization, enabling biological and industrial applications.

GFC uses either microporous with different cur-off of Mw (Figure 4C) or anion exchange resins (Figure 4D) as column packing materials. For example, using gel permeation chromatography on a Sepharose 6B column, a purified and homogeneous polysaccharide fraction with a yield of 6.25% was obtained from the solution containing crude polysaccharides extracted from *R. albonigra* (11). Additionally, the versatility of the Sepharose 6B column for GFC was further demonstrated, as it could be employed to isolate two distinct polysaccharide fractions with different Mw. Remarkably, the fraction with a lower Mw exhibited an exceptional capability of enhancing the immune system (64). Similarly, GFC with a Superdex 75 HR 10/30 column was employed to purify the water- and alkali-soluble polysaccharides from *R. vinosa*, which exhibited excellent anti-oxidant and hepatoprotective activities (56). Likewise, a purified fraction of polysaccharides with anti-tumor activity was obtained from the crude polysaccharides of *R. griseocarnosa* using a Sephadex G100 column (13). More recently, three galactoglucan fractions were obtained from the solution containing the crude polysaccharides extracted from *R. vinosa Lindblad* using a HiPrep 26/60 Sephacryl S-500 HR column, with each of the fractions exhibiting distinct immunostimulatory activities (27). Therefore, GFC is a crucial method for purifying and fractionating the polysaccharides from *Russula*, allowing the isolation of specific fractions with varying Mw. This is essential for effectively exploring the bioactivities of the polysaccharides and understanding their potential applications.

GFC is a valuable technique for purifying polysaccharides, offering size-based separation and preserving bioactivity. Its versatility allows for using columns of various sizes and different matrices. However, it also possesses certain drawbacks. It can be time-consuming, mainly for large-scale purification, and may not provide a high resolution when separating polysaccharides of similar Mw, leading to coelution. Scaling up can also be challenging due to the limited capacity of the columns. Hence, further research is warranted to improve GFC for better applicability in the purification of polysaccharides. Optimization of the technique by the correct choice of column type, matrix, and elution buffer, could enhance the resolution and efficiency. Combining GFC with other purification techniques, like ion exchange or affinity chromatography, may also improve purity and yield.

Generally, the polysaccharides extracted from mushrooms possess colored impurities due to natural pigments, environmental contaminants, cellular components, and oxidation reactions occurring during extraction (65, 66). These impurities can affect the purity and appearance of the extracted polysaccharides, making the accurate analysis and characterization of their properties challenging. Moreover, they can impact the bioactivity or functionality of the polysaccharides and hence, may interfere with subsequent applications. Thus, a purification step for decolorization is necessary to remove these impurities and obtain a refined and colorless polysaccharide. Several decolorization methods, such as activated carbon- and chitosan-based adsorption, H_2_O_2_-based degradation, microporous resin-based adsorption, alumina-based column chromatography, polyamide-based decolorization, and static mixer-based adsorption are available. These techniques offer a range of options to effectively remove pigments and colored impurities from extracted polysaccharides, enhancing the purity and quality of the final product (67). Though decolorization has been commonly employed in various plants spp. (68, 69), its applicability in *Russula* remains underexplored. Therefore, further research in this area is vital to obtain pure polysaccharides from *Russula* and unlock their full potential for various bioactivities.

## 4 Bioapplications of polysaccharides from *Russula* based on their bioactivities

*Russula* is a mushroom renowned for its rich content of bioactive compounds, including proteins, polysaccharides, phenols, lectins, and terpenoids. Hence, it is valued as a source of traditional food due to a high carbohydrate content occurring in the cell wall and the mycelium (70). The polysaccharides derived from *Russula* demonstrated multiple beneficial effects. For example, the applicability of *Russula-*derived polysaccharides has allowed the transition of *Russula* beyond its traditional role as a food source to harness its bioactive functionalities, thereby offering promising prospects for use in various fields.

Recent studies have been provided compelling evidence supporting the exceptional bioactive properties of polysaccharides derived from *Russula,* such as anti-oxidant, anti-tumor, immune-system-modulating, anti-inflammatory, and anti-bacterial activities (71–73). Therefore, this review aims to summarize the diverse bioactive properties exhibited by *Russula*-derived polysaccharides, shedding light on their immense potential for applicability in medicine and different bio-fields.

### 4.1 Anti-oxidant activity

Antioxidation fundamentally involves countering the detrimental effects of free radicals, produced as natural byproducts of normal metabolic processes occurring within the body (32, 74). However, excessive production of free radicals can lead to the accelerated depletion of nutrients, overburdening the anti-oxidant system of the body and disrupting the metabolic equilibrium, thereby contributing to oxidative-state-related stress and various ailments such as cardiovascular diseases, accelerated aging, rheumatoid arthritis, and cancer (75). Thus, the vital importance of anti-oxidants in maintaining the overall well-being of an organism is underscored.

**Figure 5:**
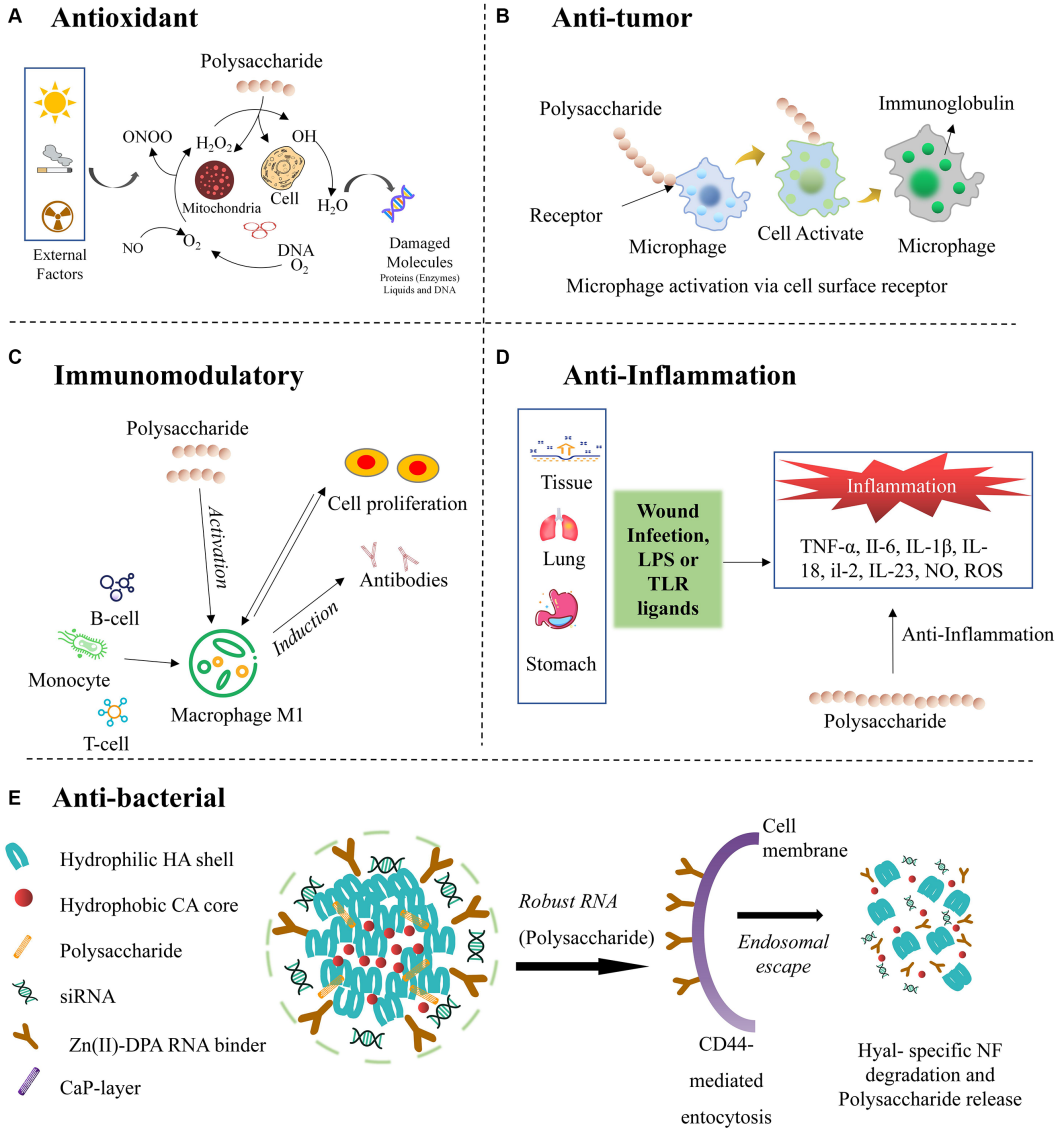
Bioactive properties and mechanism of action of the polysaccharides from *Russula*.

In many eukaryotes, oxidation primarily occurs within the mitochondria, which play a central role in controlling the apoptotic signals in nonalcoholic fatty liver disease (6, 76). Increased oxidation within the mitochondria leads to a rise in ATP flux and enhances the Kreb’s cycle, producing reactive oxygen species (ROS), which including different free radicals. Consequently, reducing the ROS levels by anti-oxidants of the polysaccharide becomes essential for the normal functioning of the organism by repairing DNA (31), which can be seen in schematic diagram in Figure 5A.

Notably, *Russula*-derived polysaccharides has been identified with crucial role as anti-oxidants in scavenging free radicals in organisms and hence inhibited hepatic steatosis, prevented inflammation, and regulated lipid metabolism (77). For example, it is reported that polysaccharides extracted from *R. griseocarnosa* with highest yield possessed a maximum anti-oxidant activity of 6 mg/mL, which showed a remarkable scavenging capacity of 85% for DPPH free radicals, and a significant scavenging capacity of 71% for ABTS free radicals, indicating a concentration-specific ability of the hydroxyl radicals (•OH) scavenging (31). In addition, the isolation of polysaccharide fractions enriched with β-glucans (RuseHap) from the fruiting bodies of *R. senecis* has been studied, which demonstrated significant anti-oxidant activity, exhibiting potent scavenging capabilities for the radicals such as hydroxyl, 2,2-diphenyl-1-picrylhydrazyl, and 2′-azinobis (3-ethylbenzothiazoline-6-sulfonic acid), and notable chelating and reducing abilities, with an effective half-maximal concentration ranging from 225–2,909 μg/mL (78). In addition, the water-soluble and alkali-soluble polysaccharides extracted from *R. vinosa* exhibited potent anti-oxidant activities, including DPPH radical and H_2_O_2_ scavenging, inhibition of lipid peroxidation, and Fe^2+^ chelating activity, administration of which significantly prevented CCl_4_-induced liver damage in mice through enhancing the activation of anti-oxidant enzymes and reduced malondialdehyde levels, indicating their hepatoprotective effects (56). Based on the aforementioned works, it can be known that the *Russula*-derived polysaccharides as effective natural compounds have demonstrated their significant potential in combating oxidative-state-related stress, exhibiting impressive anti-oxidant activity, scavenging various free radicals, and reducing the damage caused due to oxidative-state-related stress.

Furthermore, chemical modifications of polysaccharides have also been proposed to enhance their anti-oxidant activity and even imparted novel functional properties, making them promising candidates for applicability in diverse fields. For example, esterification of polysaccharides from *R. alutacea* Fr. with phosphoric acid resulted in the production of water-soluble phospho-polysaccharides, which exhibited a higher content of carbohydrates contents (7.101 mg/g) compared to that of the unmodified polysaccharide (6.863 mg/g), with a significantly improved free radical scavenging activity of 81.70% compared to 74.84% of the unmodified polysaccharide; thereby indicating that the anti-oxidant activity of the polysaccharides extracted from *R. alutacea* could be improved by chemical modification with phosphoric acid (74). In addition, the enzyme-based modification also improved the anti-oxidant capacity of the polysaccharides from *Russula*. For example, the enzyme-based modification of the polysaccharides extracted from *R. alutacea* enhanced their reducing power as well as their ability to scavenge •OH radicals, superoxide anions (O_2_^−^), and DPPH radicals, but to a lesser extent than ascorbic acid (79). Overall, the enzyme- or chemical-based modifications of the polysaccharides from *Russula* had improved their anti-oxidant properties, making them potential candidates for diverse applications in medicine and as functional foods.

Currently, the emergence of innovative methodologies and advanced techniques has significantly broadened the scope of research on *Russula*-derived polysaccharides, revealing their impressive anti-oxidant activities and thus opening up promising avenues for practical applications. However, deciphering the structure-antioxidant activity correlation requires meticulous investigations to unveil the mechanisms underlying their anti-oxidant capabilities and instill confidence in their effective utilization. A comprehensive and systematic exploration, accompanied by a methodical analysis, is imperative to fully harness the vast potential of *Russula*-derived polysaccharides and their derivatives.

### 4.2 Anti-tumor activity

The current arsenal of cancer treatments, encompassing chemotherapy and radiation therapy, often leads to distressing side effects, including nausea, impairment of mental functions, and a loss of appetite (80, 81). Additionally, the emergence of resistance to chemotherapy poses a significant challenge in controlling the progression of cancer. In pursuit of more promising alternatives, research has focused on pivotal areas, notably tumor-targeted drugs, and polysaccharides with potent anti-tumor activity. Tumor-targeted drugs offer the advantage of selectively attacking cancer cells, minimizing harm to healthy tissues, and potentially mitigating the adversities associated with conventional therapies (82, 83).

Polysaccharides can exhibit anti-tumor activity due to their unique structural properties and biological interactions with the cells. Specifically, they activate the immune system, prompting the proliferation of cytotoxic cells that selectively target cancer cells (Figure 5B) (84, 85). Additionally, specific polysaccharides can directly induce apoptosis or inhibit the growth and proliferation of tumor cells. Moreover, they can disrupt angiogenesis, impeding the formation of new blood vessels crucial for tumor nourishment. These multifaceted mechanisms highlight the potential of polysaccharides as promising agents in cancer treatment and prevention, opening new avenues for therapeutic research and drug development (86).

Currently, various studies have explored the mechanisms behind the anti-tumor activity of *Russula*-derived polysaccharides, which indicated that these polysaccharides hindered the proliferation of cancer cells and stimulated the macrophages through receptor-mediated and endocytosis-mediated activation. These findings highlight the potential of *Russula*-derived polysaccharides as valuable candidates in cancer research and the development of novel therapeutics. For example, the structure and composition of the polysaccharides extracted from *R. edodes* (PRG-1) were analyzed using ultraviolet and infrared spectroscopy. Then, it is found that PRG-1 showed ability to induce apoptosis and effectively inhibited the growth of HeLa and SiHa tumor cell lines, with a positive correlation observed between the concentration of *Russula* polysaccharides and the number of apoptotic cells, indicating their ability to induce apoptosis in tumor cells and suppress tumor growth, thereby confirming their potent anti-tumor activity (13). Similarly, the anti-tumor activity of the polysaccharides extracted from *R. virescens* (RVP) determined using the MTT method and Caco-2 cancer cells can be enhanced when it was chemically modified using sulfuric acid in comparison to the unmodified RVP. These results indicated both original and modified polysaccharides from *Russula* possess the potential as valuable, biomedical, anti-tumor agents (14). Currently, well-established, anti-tumor mechanisms of polysaccharides extracted from different plants included direct mechanisms, such as apoptosis induction and metastasis inhibition, and indirect defense mechanisms of an enhancement in the T-cell-based immune response (87).

Overall, *Russula* polysaccharides have shown potential promise as potential anti-tumor agents due to their advantageous properties, such as inhibiting cancer cell proliferation and activating macrophages through receptor-mediated and endocytosis-mediated mechanisms. In addition, the advantages of *Russula* polysaccharides as anti-tumor agents and the ongoing research to overcome the current limitations offer promising prospects for their future development and application in the anti-tumor field. Therefore, further investigations are warranted to elucidate the potential anti-tumor activities of polysaccharides from *Russula* and their therapeutic applications in cancer treatment. However, it should be pointed out that future work should focus on exploring and elucidating the detailed pathways involved in their anti-tumor effects to optimize their application as effective cancer therapies.

### 4.3 Immunomodulatory activity

The rising prevalence of cancer, immuno-deficiency disorders, and infectious diseases has underscored the increasing significance of immunotherapy, which aims to augment the immune mechanisms of humans (88, 89). Immunity entails recognizing and repel invaders, thereby enhancing the immune system in a regulated manner and fortifying the defense response of the host. Generally, immune-related functions serve as the primary line of defense against attack by pathogens, playing a crucial role in preserving the stability between the internal and external environments and protecting against pathogens and immune-related threats.

In the structure of polysaccharides, sugar residues are mainly connected by 1,6→, 1,2→, 1,3,6 → glucoside bonds, and have a unique three helical structure, which gives it immunomodulatory. It can therefore be used as a natural source of immunomodulatory. These complex carbohydrates have diverse chemical compositions, allowing them to interact with the immune cells and cell receptors (9, 64). Polysaccharides can specifically stimulate or regulate immune responses by promoting the production of cytokines and other immune mediators. The activated immune cells, like macrophages and dendritic cells, can enhance phagocytosis and antigen presentation. Additionally, polysaccharides can modulate the functions of T- and B-cells, influencing their proliferation, differentiation, and ability to produce antibodies, further promoting innate and adaptive immune responses (Figure 5C) (15, 16). Hence, a natural origin, excellent biocompatibility, and low toxicity contribute to their utility as immunomodulatory agents, with potential benefits in enhancing immune responses and combating various diseases, including cancer and infections by pathogens. The wide range of polysaccharides derived from different sources, such as mushrooms, plants, and marine organisms, offer versatile options for disease management and promoting human health.

In general, the structure of *Russula*-derived polysaccharides exhibits a high diversity owing to variations in their compositions, conformations, Mw, and other properties, which significantly influence their bioactivities of immunomodulatory activity (32). A water-soluble heteroglycan (PS-II) was isolated from the aqueous extracts of an ectomycorrhizal edible mushroom of *R. albonigra* exhibited an *in vitro* activation of macrophages through the production of NO, as well as the proliferation of splenocytes and thymocytes, suggesting its potential as an immunostimulatory agent (64). In addition, Nandi et al. (89), found that polysaccharides from *Russula albonigra* (Krombh.) Fr. PS-I by hot water extraction could increase the amounts of macrophages in the immune system with the increased dose of PS-I. For example, they reported that the optimal production of macrophages with an amount of per 5 × 10^5^ could reach 24.5 μM NO under 100 μg/mL PS-I. Concretely, a certain concentration of PS-I can be considered an effective macrophage stimulant, indicating that PS-I has a specific immune stimulation effect on macrophages. In addition, Khatua et al. (78) found that macrophage of TLR-2 and TLR-4 have potential mutual stimulation when stimulated by viral nucleic acid components, which is due to its ability to recognize the polysaccharide from *Russula senecis* (RuseHap). In addition, nuclear factor-κB (NF-κB) can also be activated by the obtained polysaccharide, which in turn promotes the release of cytokines for improving immune function. The aforementioned results reveal that polysaccharide isolated from *Russula* has the ability to promote the upregulation of the expression of specific cytokines and to stimulate the secretion of cytokines by immune cells in an immunomodulatory role.

Overall, the potential applications of polysaccharides derived from *Russula* as promising natural immunostimulatory with potential applications in the food and pharmaceutical industries are highlighted, indicating their promising role in various applications for health and therapeutic purposes. However, certain limitations and challenges still need to be addressed in the future. For example, a deeper understanding of the mechanisms underlying their immunomodulatory effects is essential to optimize their applicability as immune-related agents at the molecular and cellular levels. Additionally, the development of formulations and safety assessments can enhance their efficacy, bioavailability, and safety as promising immunomodulatory therapies for managing various immune-related disorders.

### 4.4 Anti-inflammation activity

The incidence of cardiovascular diseases, diabetes, obesity, and atherosclerosis has been intricately linked to the underlying metabolic disorders and subsequent inflammation. The deleterious impacts of these inflammatory factors not only affect the structural or functional integrity of various tissues and organs but may also facilitate the proliferation of tumor cells (17, 90). Consequently, there is a pressing need to explore novel anti-inflammatory drugs derived from natural sources, ensuring safety and efficacy, to address this critical healthcare challenge.

Polysaccharides have emerged as promising anti-inflammatory agents owing to their distinct structural and functional characteristics. Through interactions with immune cells and immune system-related signaling pathways, polysaccharides can effectively downregulate the production of pro-inflammatory cytokines and mediators while concurrently promoting the production of anti-inflammatory cytokines like TNF-α, II-6, IL-1β, IL-18, and IL-23 (Figure 5D). Moreover, their versatility as natural compounds derived from various sources, such as mushrooms, seaweeds, and plants, with low toxicity and minimal side effects, underscores their potential therapeutic value (15, 91) As these polysaccharides offer a multifaceted approach to modulating immune responses, they present an attractive avenue for managing inflammatory conditions and related diseases. Hence, various efforts were made to extract the polysaccharides from *Russula* and optimize their applicability as effective anti-inflammatory agents.

Recently, Khatua et al. (78) found that the water-soluble polysaccharides obtained from *R. senecis* demonstrated immune-stimulatory effects, especially on murine macrophages. Specifically, treatment with these polysaccharides demonstrated a substantial upregulation of TLR-4 and 2 and nuclear factor κB (NF-κB) in murine macrophages, leading to reduced levels of cyclooxygenase-2 (COX-2), inducible nitric oxide synthase (iNOS), tumor necrosis factor-α (TNF-α), IκB-α, and interferon-γ (IFN-γ) associated with the immune system. Meantime, all of TLR-4, TLR-2, NF-κB, COX-2, iNOS, TNF-α, IκB-α, and IFN-γ are the important cytokines in the TLR2/TLR4/NF-κB pathway, which is closely related to immune and inflammatory responses. Generally, polysaccharides can be recognized by TLR2/TLR4 receptors on the cell surface and directly promote/inhibit the expression of mRNAs associated with immune and inflammatory responses, affecting the production of these cytokines at the transcriptional level. Therefore, the specific immune stimulation effect of *Russula* polysaccharides on macrophages can up-regulate TLR-2, TLR-4, NF-κB and down-regulate COX-2, iNOS TNF-α, IκB-α, and IFN-γ. In addition, a polysaccharide with an average Mw of 1029.7 kDa was purified from *Russula*, which remarkably downregulated the expression of NF-κB, iNOS, and COX-2 in macrophages. In the larvae of zebrafish (*Danio rerio*), the polysaccharides exhibited a striking ability to reduce ROS and O_2_^−^ levels induced by LPS, effectively restoring the normal heart function against inflammation- and oxidative-state-related damage (17). Based on these works, it can be known that the dynamic influence of polysaccharides on the stimulation of macrophages reveals a remarkable interplay, where these potent signaling molecules orchestrate their actions (15). Hence, *Russula*-derived polysaccharides have emerged as promising natural supplements, holding great potential for applicability in anti-inflammatory formulations.

Overall, considerable attention has been devoted to exploring the remarkable nutritional and anti-inflammatory potentials of *Russula*-derived polysaccharides. The positive correlation between these and cytokine levels underscored its valuable role. The stimulation of the proliferation of lymphocytes and production of antibodies by the anti-inflammatory cytokines represent a pathway effective for the reduction of inflammation, further highlighting the significance of *Russula*-derived polysaccharides in promoting the overall health of humans.

### 4.5 Anti-bacterial activity

Several pathogenic bacteria and viruses occur in nature. The escalating number of cases of bacterial resistance poses a growing concern, which is exacerbated by the abuse of anti-bacterial drugs or antibiotics, leading to fungal infections, transfer of resistance-related genes, and widespread consequences (92). As a result, attempts have been made to extract novel natural products as anti-bacterial agents, heralding a new frontier in this field of research. Abundant natural sources of anti-bacterial agents present a treasure trove for medicinal exploration (93). The naturally occurring anti-bacterial drugs used either directly as whole plant material or after extraction from plants boast remarkable advantages, including heightened efficacy, minimal toxicity, infection-free properties, and scarce side effects, all complemented with excellent biocompatibility. Consequently, their popularity continues to surge, resulting in widespread applications. Polysaccharides can inhibit bacterial adhesion, disrupt cell membranes, and degradation of cell membranes necessary for bacterial growth (Figure 5E). Additionally, specific polysaccharides can inhibit bacterial enzymes and promote the production of antimicrobial compounds (12, 94). These diverse mechanisms of action make them valuable anti-bacterial agents, offering potential applicability as stand-alone treatments or in combination with traditional antibiotics.

Exciting advancements in harnessing the potential of *Russula*-derived polysaccharides are being made. Notably, they lack cell toxicity, rendering them ideal additives for complementation with traditional anti-bacterial agents. This groundbreaking approach holds immense promise, presenting a safe and potent alternative that could revolutionize health and overall well-being (95). Generally, the *in vitro* antibacterial activity of *Russula*-derived polysaccharides may be affected by their structural characteristics of degrees of substitution (DS), molecular weight (Mw), and chain conformation. Specifically, polysaccharides can interact with bacteria through hydrophilic and hydrophobic interactions, electrostatic adsorption, or the presence of sugar receptors, which is related to the characteristics of polysaccharides. In addition, the polysaccharides can perform the antibacterial ability by increasing the permeability of cell membranes, inhibiting the adsorption of pathogenic bacteria to host cells, and blocking the transmembrane transport of nutrients or energy substances (6). For example, Li et al. (14) successfully introduced sulfate groups into *Russula Virescens*-derived polysaccharide RVP (SRVP), which could effectively improve the antibacterial ability against *Escherichia coli* and *Staphylococcus aureus*. In addition, it showed that the minimum inhibitory concentration (MIC) of SRVP for *Escherichia coli* and *Staphylococcus aureus* were 4 mg/mL and 3 mg/mL, respectively. In addition, it is found that the zones of inhibition of SRVP against *Staphylococcus aureus* and *Escherichia coli* were 20.5 ± 0.09 mm and 16.7 ± 0.05 mm by Disc diffusion analysis, respectively (94). Generally, a lower MIC value and higher zones of inhibition value for polysaccharides indicate better antibacterial activity. Based on the aforementioned discussion, it can be known that polysaccharide derived *Russula viresens* has higher antibacterial activity against *Staphylococcus aureus* compared to *Escherichia coli*. In addition, it is also indicated that polysaccharide derived from *Russula virescens* has stronger antibacterial activity against gram-positive bacteria, highlighting their promising potential as effective antimicrobial agents.

Generally, the cell wall of Gram-positive bacteria is a monolayer containing lipopolysaccharide, while the cell wall of Gram-negative bacteria is multi-layered with a low thickness and lacking lipopolysaccharide (96). The thick peptidoglycan layer of Gram-positive bacteria easily absorbs foreign substances, including antibiotics. Additionally, Gram-positive bacteria have higher cell membrane permeability, making them more readily penetrated compared to Gram-negative bacteria. Thus, the variations in cell membrane composition and structure make Gram-positive bacteria susceptible to polysaccharides. When exposed to polysaccharides, Gram-positive bacteria undergo changes in their cell membranes, which can ultimately impact their nutrient and energy transport, thereby affecting the bacteria’s ability to defend against the polysaccharides. Moreover, the sulfated polysaccharides derived from *R. virescens* have been found to effectively combat the growth of *Escherichia coli*, primarily due to a magnification of the intrinsic anti-bacterial potency of the unmodified polysaccharides (14). Specifically, sulfonic acid groups (–SO_3_H) can be incorporated into polysaccharide molecules to replace the hydroxyl group (–OH) after sulfonation (97). This modification enhances the affinity between the sulfated polysaccharide from *Russula virescens* and bacterial receptors, improving its recognition and binding ability by altering the spatial structure and conformation of the polysaccharide (98). Simultaneously, the sulfation modification increased the charge density, solubility, and stability of the sulfated polysaccharide, allowing for improved dispersion and diffusion in the bacterial environment, thus enhancing its ability to inhibit bacterial growth. Additionally, the modification improved the specificity and selectivity of the polysaccharide, enabling it to act more targetedly on specific bacteria (99), such as *Escherichia coli and Staphylococcus aureus*. This suggests that sulfation modification can be used to enhance the antibacterial activity of various polysaccharides derived from plants. However, the current research landscape solely encompasses the evaluation of the anti-bacterial capacity of the *Russula*-derived polysaccharides, leaving ample room for further exploration. Additional studies should be undertaken to expand the understanding of their anti-bacterial potential, paving the way for fascinating discoveries and potential applications in this field.

In summary, the polysaccharides extracted from *Russula* spp. are non-cytotoxic and display promising anti-bacterial activity, opening exciting avenues for exploring their potential applicability as *in vivo* anti-fungal agents. Future investigations should delve into the structural elucidation of the active compounds and unravel the intricate mechanisms at play. Through continued research, the complete potential of these polysaccharides can be unlocked, and their remarkable properties harnessed for advancing the field of anti-bacterial therapeutics.

This review comprehensively summarizes the diverse bioactivities of *Russula*-derived polysaccharides, including anti-oxidant, anti-tumor, immunomodulatory, anti-inflammatory, and anti-bacterial activities. An overview of these activities and their profiles are compiled in Table 1, encapsulating the wealth of the research undertaken and highlighting the multifaceted potential of *Russula*-derived polysaccharides in various fields. Notably, the polysaccharides derived from *Russula* also harbor additional potential, such as anti-ulcer, anti-diabetic, and anticoagulant abilities, which are yet to be fully explored. Therefore, further research is imperative to fully unravel the potential of *Russula*-derived polysaccharides, which can broaden their applicability in enhancing human health.

**Table 1:** Bioactivities and applications of *Russula*-derived polysaccharides.

Biological activity	Source	Activity capacity	Applications	Reference
Anti-oxidant	*Russula griseocarnosa*	PRG showed a higher superoxide radical scavenging activity, with a maximum of 75% at 6.0 mg/mL	Anti-cervical carcinoma cells Hela and Siha	(31)
	*Russula vinosa* Lindblad	Radical scavenging activity (IC_50_ = 1.55 ± 0.04 and 3.37 ± 0.21 mg/mL, respectively)	Antioxidation and hepatoprotective effects against acute liver injury in mice	(53)
		Hydrogen peroxide scavenging activity (IC_50_ = 6.07 ± 0.24 and 9.23 ± 0.54 mg/mL, respectively)		
	*Russula alutacea* Fr.	The scavenging of DPPH, OH, O_2_ and the reduction ability for phosphoric esterification	Phosphoric esterification of polysaccharides	(71)
	*Russula alutacea*	The EC_50_ values of ascorbic acid, unmodified, and modified polysaccharides were 0.573 mg/mL, 1.26 mg/mL, and 1.14 mg/mL, respectively	Improved the clearance rate of modified polysaccharides	(76)
Anti-tumor	*Russula griseocarnosa*	—	As a natural inhibitor of tumor cell proliferation in cervical carcinoma	(13)
	*Russula virescens*	The highest inhibition rate of 11.1% was obtained at 250 mg/mL	Sulfated polysaccharides provided new insights into cancer prevention and treatment	(14)
Immunomodulatory	*Russula vinosa* Lindblad	—	Promote macrophage proliferation, phagocytosis, and the release of nitric oxide and cytokines (TNF-α and IL-1β)	(27)
	*Russula alatoreticula*	Triggering the transcriptional activation of Toll like receptor-2 (TLR-2), TLR-4, nuclear factor κB (NF-κB), cyclooxygenase-2 (COX-2), inducible nitric oxide synthase (iNOS), tumor necrosis factor-α (TNF-α), Iκ-Bα, interferon-γ (IFN-γ) and interleukin-10 (IL-10) encoding genes	The increased role of *R. alatoreticula* in the stimulation of innate immunity	(32)
	*Russula albonigra* (Krombh.) Fr.	*In vitro* activation of macrophages by the production of nitric oxide (NO) and the proliferation of splenocytes and thymocytes	Be used as immunoenhancing and anti-tumor materials	(61)
Anti-inflammation	*Russula senecis*	High affinity for Fe^2+^ and an instant ability to donate electrons with EC_50_ values ranging from 80–3,885 μg/mL	In the preparation of dietary supplements for enhancing general health	(15)
	*Russula alutacea* Fr.	Suppressed the morphological changes in cells and inhibited the production of NO in LPS-induced RAW 264.7 cells extracellularly and intracellularly	Ameliorated oxidative-state-related stress and mitochondrial dysfunction; a potential functional resource for protecting against inflammatory and oxidative damage	(17)
Anti-bacterial	*Russula alatoreticula*	Fe^2+^ chelating and reducing power with EC_50_ ranging from 785–2,500 mg/mL	Representing *R. alatoreticula* as a value-added bio-resource	(6)
	*Russula virescens*	MIC and MBC values of SRVP1–20 against *Escherichia coli* were 4 and 5 mg/mL, and against *Staphylococcus aureus* were 3 and 5 mg/mL, respectively	As natural, alternative derivatives for industrial and biomedical use	(14)
	*Russula xerampelina*	*Russula xerampelina* for the control of phytopathogens	For the control of phytopathogens	(95)

### 4.6 Other bioactivities

Currently, the anti-ulcer, anti-diabetic, and anticoagulant properties of polysaccharides and other extractives from different sources have been explored. Gastric ulcer is one of the most diseases for humans. Various works have been conducted to extract effective ingredients from natural plants as anti-ulcer agents. For example, the extracted polysaccharides from *Lentinus squarrosulus* possessed the anti-ulcer ability, which showed a significant protective effect on perfecting the gastric mucosa of astragalus rats and reducing pro-inflammatory cytokines (100). Therefore, according to the similar characteristics of polysaccharides *Russula* and *Lentinus squarrosulus*, it is worth investigating the anti-ulcer ability of *Russula*’s polysaccharides.

Diabetes mellitus is a metabolic disease characterized by hyperglycemia caused by multiple causes. It is required lifelong medication for the patient as there is no cure for diabetes. For the patient, many complications can be caused if the level of blood sugar is not well controlled. Hence, controlling the level of blood glucose is one of the necessary means to prevent diabetes. Currently, inhibiting the key enzyme in the digestive system with different medicines and health products is the focus of diabetes prevention. It has been reported that the extractives from *Ganoderma lucidum* could reduce blood glucose levels by inhibiting the carbohydrate hydrolases of α-glucosidase and α-amylase (101). It has been reported that the extractives of polysaccharides from *Russula* could inhibit the activity of α-glucosidase (102). Hence, it is speculated that the extracted polysaccharide from *Russula* may show good anti-diabetic performance as a functional food.

Anticoagulation is the process of stopping blood clotting by interfering with specific coagulation factors. Currently, various anticoagulant medications have been used, such as heparin, hirudin, and calcium ion-chelating agents. It has been identified that marine fungi and algae are the primary natural resource to obtain anticoagulants. Thimmaraju et al. (102) found that anticoagulant of HUP-2 could show the anticoagulant activity by blocking the breakdown of fibrin in the blood, which can be affected by carbohydrates. Hence, it is speculated that the polysaccharide from *Russula* may help to reduce the occurrence of blood coagulation in humans for inhibiting thrombotic diseases.

Based on the aforementioned discussion, it can be known that the polysaccharide from *Russula* possesses anti-ulcer, anti-diabetic, and anticoagulant properties. However, there a rare works that focus on this state-of-the-art application of polysaccharide from *Russula in* these bio-fields, which should be further explored.

## 5 Conclusions and perspectives

The polysaccharides derived from *Russula* are a treasure trove of valuable bioactive components, fostering human health with their diverse roles, minimal cytotoxicity, exceptional safety, and naturally pure essence. The bioactivities of *Russula*-derived polysaccharides include anti-oxidant activity, where modified polysaccharides from *Russula* demonstrate improved properties in repairing DNA and reducing levels of ROS. Polysaccharides also showed anti-tumor activity by inducing apoptosis or inhibiting the growth and proliferation of tumor cells, as well as disrupting angiogenesis. Moreover, they can modulate immune responses, stimulate the production of cytokines, and modulate the functions of immune cells, suggesting applications in viral infections, immune system disorders, and drug treatments. Additionally, modification of the *Russula*-derived polysaccharides can exhibit anti-inflammatory and anti-ulcer effects, which is based on its anti-oxidant activity. Furthermore, they have shown potential anti-diabetic and anti-bacterial activities, inhibited bacterial adhesion and enzymes and held promise as antifungal agents and antibacterial biomaterials. These unique attributes make them a valuable resource with immense potential candidates for various applications in medicine, such as antioxidant therapy, cancer treatment, modulation of immune responses, and anti-inflammatory and anti-ulcer treatments. The diverse mechanisms of action and advantageous properties of these polysaccharides indicate their potential applicability as stand-alone treatments or in combination with traditional therapies, offering versatile solutions for various health conditions and potential development of novel functional food products.

Although previous trials have demonstrated the successful applications of *Russula*-derived polysaccharides, the sources and precise mechanisms of their bioactivities remain partly elusive, providing promising avenues for future work. Investigating the underlying mechanisms, structure-activity relationship, and synergistic effects can optimize their therapeutic potential. Preclinical and clinical studies are essential to evaluate their safety and efficacy, while the development of formulations and biotechnological approaches can enhance their stability and industrial-scale production. Exploring their applicability as foods and nutraceuticals, addressing antimicrobial resistance, and conducting toxicological and environmental impact studies are critical steps toward realizing their full potential in sustainable practices and promoting human health. Thus, the *Russula*-derived polysaccharides can emerge as valuable bioactive agents with diverse applications in the pharmaceutical and food industries.

## Author contributions

YC: Writing – original draft. JG: Writing – original draft. BY: Writing – original draft. PW: Writing – review & editing. HW: Writing – review & editing. CH: Supervision, Writing – review & editing.
